# Current Advances in Nanotechnology-Mediated Delivery of Herbal and Plant-Derived Medicines

**DOI:** 10.34172/apb.2023.087

**Published:** 2023-07-19

**Authors:** Amir Jalili, Rafieh Bagherifar, Ali Nokhodchi, Barbara Conway, Yousef Javadzadeh

**Affiliations:** ^1^Department of Pharmaceutical Technology, Faculty of Pharmacy, Eastern Mediterranean University, Famagusta, North Cyprus.; ^2^Student Research Committee, Tabriz University of Medical Sciences, Tabriz, Iran.; ^3^Department of Pharmaceutics, Faculty of Pharmacy, Tabriz University of Medical Sciences, Tabriz, Iran.; ^4^Pharmaceutics Research Laboratory, School of Life Sciences, University of Sussex, Arundel Building, Brighton BNI 9QJ, UK.; ^5^Lupin Research Center, Coral Springs, Florida, USA.; ^6^Department of Pharmacy, School of Applied Sciences, University of Huddersfield, Huddersfield, UK.; ^7^Institute of Skin Integrity and Infection Prevention, University of Huddersfield, Huddersfield, UK.; ^8^Biotechnology Research Center, and Faculty of Pharmacy, Tabriz University of Medical Science, Tabriz, Iran.

**Keywords:** Phytomedicine, Herbal drug, Nanotechnology, Drug delivery systems, Nanophytomedicine

## Abstract

Phytomedicine has been used by humans since ancient times to treat a variety of diseases. However, herbal medicines face significant challenges, including poor water and lipid solubility and instability, which lead to low bioavailability and insufficient therapeutic efficacy. Recently, it has been shown that nanotechnology-based drug delivery systems are appropriate to overcome the above-mentioned limitations. The present review study first discusses herbal medicines and the challenges involved in the formulation of these drugs. The different types of nano-based drug delivery systems used in herbal delivery and their potential to improve therapeutic efficacy are summarized, and common techniques for preparing nanocarriers used in herbal drug delivery are also discussed. Finally, a list of nanophyto medicines that have entered clinical trials since 2010, as well as those that the FDA has approved, is presented.

## Introduction

 Phytomedicines also called herbal medicines, are mixtures of plant metabolites containing pharmacologically active compounds with some healing and therapeutic properties. due to the benefits such as fewer adverse effects and low cost, herbal medicines have been used since ancient times as therapeutic agents in various diseases. In addition, over one-third of all FDA-approved new molecular entities are natural products and their derivatives.^1,2^ The first plant-derived drug was painkiller morphine, with a mechanism of inhibiting the discharge of neurotransmitters from presynaptic neurons and was authorized for utilization in 1827.^3^ Later, many other products were developed, including paclitaxel, which is used today as an anticancer agent in ovarian, breast, lung, and other cancers and extracted from the pacific yew plant (*Taxus brevifolia*).^4,5^

 The significant steps to obtain herbal extracts or oils from plant materials generally include harvesting (to suppress plant metabolism at the right time), drying (to protect the active substance by inhibiting enzymes), size reduction (to increase the surface area and thus the improvement of solvent extraction) and extraction (in order to obtain therapeutic portion and omission of inert parts). Finally, the resulting extract can be traditionally formulated in various dosage forms such as solid, liquid, and semi-solid, or encapsulated in novel drug delivery systems such as liposomes, pyrosomes, polymeric NPs, etc.^6-8^

 Despite the prominent pharmacological actions of herbal drugs in various diseases, several challenges, including pharmacokinetic drawbacks such as low bioavailability and limited absorption and physicochemical challenges like poor water and lipid solubility, large molecular size, and instability, can reduce their efficacy, primarily upon oral administration.^9,10^ An effective drug delivery system is needed to overcome the abovementioned barriers, reduce repeated administration, and increase patient compliance.^11^

 In recent decades, nanotechnology-based delivery systems have received much attention in phytomedicine. The encapsulation of herbal drugs in nanocarriers and overcoming the above-mentioned limitations provides benefits such as improved solubility, protection from degradation, reduction of side effects, controlled release, and consequently optimal bioavailability and therapeutic efficacy.^12-14^

 This review outlines the challenges of phyto/herbal medicines, including physicochemical and pharmacokinetic drawbacks. Different types of nanocarriers are also discussed as novel and efficient strategies in herbal drug delivery with the potential to overcome the above-mentioned challenges. Some of the common techniques used for the formulation of nanoparticles (NPs) have been reviewed. Therefore, an overview of FDA-approved nanophytomedicines as well as those being used in clinical trials since 2010, has been provided.

## Herbal medicines: Challenges

 Herbal medicines are a mixture of various ingredients with different physicochemical properties.^15^ In addition, poor gastrointestinal (GI) absorption and consequent low oral bioavailability of herbal drugs are due to various factors, including high molecular weight, poor solubility in GI fluids, limited permeability through cell membranes, degradation in the GI tract, hepatic presystemic metabolism, and P-glycoprotein (P-GP/MDR1/ABCB1)]-mediated gut efflux.^16,17^ Therefore, the development and preparation of herbal formulations face various challenges.

 Nanotechnology-based techniques have been developed to overcome the above-mentioned limitations and increase the bioavailability of herbal medicines.

## Nanotechnology for herbal drug delivery

###  The importance of nanotechnology

**Figure 1:**
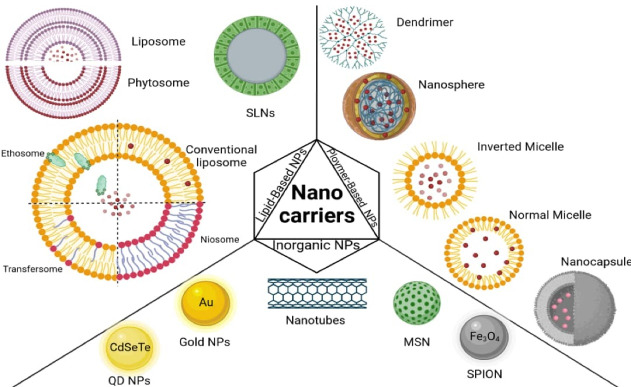
Schematic representation of common nanocarriers for herbal drug delivery

 Nanotechnology can be used to develop products with novel and improved actions and physicochemical properties particularly in the medical field.^18^ Nanocarriers protect their payload from degradation, improve bioavailability, reduce the therapeutic dose and side effects, and provide targeted therapy and controlled release of phytomedicine.^19-21^ Different classes of nanocarriers, including lipid-based NPs, polymer-based NPs, and inorganic NPs, have been used for drug delivery in phytomedicine, which will be discussed in detail below. A schematic of common nanocarriers is shown in Figure 1.

###  Lipid-based nanocarriers for herbal drug delivery

 In addition to the benefits mentioned in the previous section, lipid-based NPs such as solid lipid nanoparticles (SLNs), liposomes, and phytosomes also have the advantages of biocompatibility and the ability to improve the aqueous solubility of poorly soluble herbal drugs.^22^ Lipid-based nanocarriers are prepared using various materials and methods depending on their target. Challenges like scale-up and physical instability such as aggregation must be considered in the choice of preparation method.^23^ Following the preparation of NPs, parameters such as size, morphology, and surface properties should be determined because they play an essential role in the cellular uptake and pharmacological effects of NPs.^24^

 Liposomes are vesicular NPs which consist of concentric lipid bilayers made of amphipathic phospholipid molecules that assemble to create spherical structures in aqueous media and surround part of the solvent.^25^ In addition to increasing the solubility of the loaded drug, the liposome has been considered as a suitable carrier in herbal delivery in terms of its ability to load both hydrophilic and lipophilic drugs besides improving bioavailability and therapeutic efficacy.^26,27^

 In 1989, an Italian pharmaceutical and nutraceutical company, Indena, successfully generated complexes of phospholipids (phosphatidylcholine) and plant actives called Phytosome^®^ and then patented the innovation.^28^ Phytosomes (refer to Figure 1), also called phytolipid delivery systems, are more stable than liposomes. Because, unlike liposomes, they have a chemical bond in their structure. Phytosomes increase the bioavailability of poorly soluble herbal medicines by increasing their absorption in GI. Some of the phytosomes comprising various phytoconstituents such as grape seed, hawthorn, Ginkgo biloba, milk thistle, ginseng, and green tea are commercialized in the USA.^29,30^

 In 1990, SLNs as colloidal NPs which containing lipids that are in solid state at room and body temperature were developed. SLNs have advantages such as excellent physicochemical stability and higher protection compared to other NPs such as liposomes and polymeric NPs. In addition, due to biocompatibility and small size (50 to 1000 nm), it is possible to use SLN herbal formulations in various routes of administration.^31,32^ Table 1 summarizes the studies performed on the most common herbal medicines loaded in lipid-based NPs in the last 5 years.

**Table 1:** A summary of lipid-based herbal nanoformulations

**Nanocarrier type**	**Active ingredients/product**	**Therapeutic activity/disease**	**Results (benefits of nanotechnology)**	**Ref.**
Liposome	Triptolide	Anticancer activity	Significant antitumor ability on breast cancer	^33^
	Curcumin	Anti-inflammatory activity	Improved antioxidant and behavioral responses in inflamed mice	^34^
		Anticancer activity	Higher therapeutic efficiency	^35^
			Significant cytotoxic effect on MCF-7 cells	^36^
			Prolonged release of curcumin Improved antitumor effect	^37^
		Anti-inflammatory activity	Prolonged release of curcumin Reduced inflammatory markers	^38^
	Capsaicin	Anticancer activity	Enhanced anticancer activity Improved pharmacokinetics properties	^39^
	Usnic acid	Antimicrobial activity	Increased antimicrobial activity	^40^
		Antimycobacterial activity	Effective antimycobacterial activity against infected macrophages	^41^
	Catechins	Anticancer activity	Significantly higher inhibition activity	^42^
		Antioxidant activity	Higher stability and antioxidant and antibacterial effects	^43^
Phytosome	Quercetin	Anticancer activity	Significantly increased apoptosis	^44^
	Naringenin	Acute lung injury	Sustained release of Naringenin Enhanced pulmonary bioavailability of Naringenin	^45^
	Silybin	Hepatoprotection activity	Higher hepatoprotection efficacy Higher drug bioavailability	^46^
	Epigallocatechin-3-gallate	Anti-Inflammatory activity	Significant anti-inflammatory activity of epigallocatechin-3-gallate	^47^
	Curcumin	Inflammation and anxiety	Reduction of adverse effects of stress on anxiety and inflammation parameters	^48^
	Ginsenosides	Antioxidant activity	Improved efficacy and bioavailability of the ginsenosides	^49^
SLN	Triptolide	Rheumatoid arthritis	Remarkable inhibition of inflammation and reduction of knee edema	^50^
		Antige + n-induced arthritis	Better therapeutic effect	^51^
	Berberine	Anticancer activity	Prolonged release of berberine	^52^
	Wogonin		Enhanced cytotoxicity Sustained and controlled release	^53^
	Epigallocatechin gallate	Antioxidant and anticancer activities	Enhanced stability	^54^
	Curcumin	Anticancer activity	Stronger cytotoxicity Higher uptake efficiency	^55^
		Pgp inhibitor	Effective reduction of the sensitivity to doxorubicin against drug-resistant TNBC tumors	^56^
		CNS diseases	Increased brain accumulation	^57^
		Anticancer activity	Increased bioavailability	^58^
		Hodgkin's lymphoma	Enhanced growth inhibitory effect	^59^
		Antioxidant activity	Improved stability	^60^
	Hibiscus rosa sinensis extract	Antidepressant activity	Greater antidepressant activity	^61^
	Myricetin	Anticancer activity	Significant increase in necrosis percentage	^62^
	Silybin	Type 2 diabetes	Enhanced absorption of silybin after oral administration	^63^
	Linalool	Anticancer activity	Higher tumor inhibitory effects	^64^

###  Polymeric nanocarriers for herbal drug delivery

 Recently, polymeric NPs have attracted more attention as a drug delivery system in phytomedicine. These NPs have a particle size of 10 to 1000 nm and are divided into two categories of nanospheres and nanocapsules based on structure. Nanospheres are polymeric matrices in which the active substance is uniformly dispersed, while nanocapsules have a core-shell structure with a polymeric shell, and the active ingredient is encapsulated in the core or is adsorbed on the polymeric membrane. Biodegradable and biocompatible synthetic or natural polymers are used to prepare polymeric NPs. These particles allow the controlled release of the drug and target it to a specific site in the body.^65-67^

 Dendrimers have been extensively studied in herbal delivery among polymers due to their unique polyvalency, monodispersity, and controllable structure.^68^ Dendrimers consist of three parts: the central core, the generations, and the terminal groups. The drug can be attached to the terminal group either covalently or non-covalently and it can be encapsulated in the hydrophobic core. Polyamidoamine (PAMAM) is the first commercialized dendrimer, which is also used to increase the absorption of poorly water-soluble drugs.^69,70^

 Polymeric micelles with a core-shell structure (10-100 nm) are another polymeric NPs that are formed by self-assembly of block copolymers consisting of both a hydrophilic block and a hydrophobic block in an aqueous medium. The hydrophobic core provides benefits such as increased solubility and protection against degradation and intracellular accumulation of the drug. The outer hydrophilic layer can achieve improved biocompatibility and active targeting. In general, the stability of polymeric micelles is higher than that of surfactant micelles.^71-73^ The studies conducted on the delivery of most common herbal medicines using different polymeric NPs during the last 5 years are summarized in Table 2.

**Table 2:** Polymer-based herbal nanoformulations

**Nanocarrier type**	**Active ingredients/product**	**Therapeutic activity/disease**	**Results (benefits of nanotechnology)**	**Ref.**
Nanospheres	Curcumin	Anticancer activity	Higher anticancer activity and apoptosis in HepG2 cells	^74^
			Increased growth inhibition and apoptosis in breast cancer cells	^75^
			Improved serum stability Enhanced apoptotic effects on tumor cells	^76^
		Skin wound healing process	Enhanced potential in cutaneous wound repair	^77^
	Berberine	Anticancer activity	Increased dissolution rate and bioavailability	^78^
	Artemether	Antimalarial activity	Sustained release of artemether	^79^
Nanocapsules	Berberine	Anticancer activity	Improved efficiency and controlled release of berberine	^80^
	Curcumin	Neuroprotective activity	Improvement in the blockade of apomorphine-induced behavioral changes	^81^
		Antimalarial activity	Controlled release of curcumin	^82^
Dendrimer	Quercetin	Antibacterial efficacy	Sustained drug release Enhanced therapeutic potential of quercetin	^83^
	Silybin	Antioxidant activity	Extended-release time and improved solubility and stability	^84^
	Curcumin	Anticancer activity	Reduction of the viability of glioblastoma cell lines	^85^
Improved antitumor effect			^86^
Polymeric micelles	Berberine	Anticancer activity	Enhanced cellular uptake and improved solubility and delivery	^87^
			Higher cellular uptake Enhanced cytotoxic effect against HCT116 cells	^80^
	10-Hydroxycamptothecin		Improved liver targeting and absorption	^88^
	Curcumin	Antibacterial activity	Enhanced penetration into the biofilms and antibacterial activity	^89^

###  Inorganic nanoparticles

 Recently, various types of inorganic NPs, such as metal NPs, mesoporous silica nanoparticles (MSNs), carbon nanotubes (CNTs), and magnetic NPs, have been used for applications in drug delivery.

 Metal NPs, the most important of which are quantum dots (QDs), gold, silver, platinum, iron (II, III) oxide, titanium dioxide, and zinc oxide, were discovered by Faraday in 1908. Recently, metal NPs have attracted attention in herbal drug delivery due to their unique properties, like the high surface area to volume ratio, many low coordination sites, the transition between metallic and molecular states, and high surface energies.^90-92^

 MSNs are capable of carrying large amounts of cargo due to their large surface area and porosity. In addition, they are widely used in both oral and parenteral drug delivery due to because of unique properties such as excellent chemical stability and biocompatibility.^93,94^

 CNTs are relatively more compatible than other inorganic NPs. These NPs, which have a tubular structure, are obtained by curling up graphite sheets and are divided into two categories: single-walled carbon nanotubes (SWCNTs) and multi-walled carbon nanotubes (MWCNTs). SWCNTs can increase the solubility and bioavailability of herbal medicines. In addition, due to their hollow structure and the possibility of surface functionalization, they play an essential role in improving the physical and chemical properties of herbal drugs.^95,96^

 Magnetic NPs are another group of inorganic NPs, among which Fe_2_O_3_ in the form of superparamagnetic NPs is not sensitive to oxidation compared to other magnetic NPs such as nickel and cobalt, so it has the potential application in biomedicine, mainly targeted drug delivery. In fact, the possibility of accumulation of magnetic NPs in the target tissue by applying an external magnetic field leads to target therapy.^97^

 The studies performed during the last 5 years on the delivery of most common herbal medicines using different types of an inorganic nanocarriers are summarized in Table 3.

**Table 3:** Inorganic NPs used in herbal nanoformulations

**Inorganic nanocarrier**	**Nanocarrier type**	**Active ingredients/product**	**Therapeutic activity/disease**	**Results (benefits of nanotechnology)**	**Ref.**
Metal NP	Gold	Berberine	Anticancer activity	Remarkable reduction of tumor weight	^98^
			Spinal cord injury	Higher anti-apoptotic and anti-inflammatory effects	^99^
		Curcumin	Anticancer activity	Higher inhibition of tumor cell growth	^100^
	Silver	Curcumin	Antibacterial activity	Improved curcumin photostability and antibacterial activity	^101^
			Carbon tetrachloride induced hepatic injury	Significant antioxidant activity	^102^
			Anticancer activity	Promoted cytotoxic effect on the tumor cells	^103^
	QD	Curcumin	Anticancer activity	Better inhibitory effect on tumor cells	^104^
MSN	folic acid–conjugated MSN	Curcumin	Antioxidant, Anticancer activity	Enhanced cellular uptake and sustained release Induction of apoptosis in vitro. Enhanced in vitro antioxidant activity	^105^
	PEGylated lipid bilayer-coated MSN	Paclitaxel and curcumin		Improved stability, solubility, and sustained release in vitro Enabled iv administration of hydrophobic drugs Promoted in vitro cytotoxic activity against breast cancer cells	^106^
Magnetic NP	Fe_2_O_3_/chitosan/montmorillonite	Quercetin	Anticancer activity	Decreased toxicity Controlled and targeted release of the quercetin	^107^
	α-Fe_2_O_3_	Sida cordifolia plant extract	Antibacterial activity	Enhanced antimicrobial activity through targeted delivery	^108^
	Fe_3_O_4_	Gallic acid	Anticancer activity	Higher anticancer activity	^109^
		Quercetin		Improved anticancer activity	^110^
	Fe_3_O_4_–β-cyclodextrin		Epilepsy disorder	Improved therapeutic efficacy	^111^
	Fe_3_O_4_	Silymarin	Anticancer activity	Higher antioxidant activity	^112^
CNT	MWCNT	Curcumin, Glycyrrhizin and Rutin	Anticancer activity	Increased stability of suspension of CNTs in aqueous media Decreased toxicity of delivery system	^113^
		Curcumin		Prolonged-release property High adsorption capacity for curcumin	^114^
	SWCNT	Curcumin		Increase in population of necrotic cells	^115^
				Improved inhibition of cancer cell proliferation	^116^
	Cancer cell membrane-modified SWCNT	Berberine		Increased accumulation in liver cancer tissue Prolonged circulation time	^117^

## Techniques used for the formulation of nanophytomedicines

###  High-pressure homogenization method

 In the high-pressure homogenization method, lipid particles are converted into nanoscale particles using high pressure and high shear stress. This method, divided into hot and cold homogenization, is widely used to produce lipid-based NPs, including emulsions, liposomes, and SLNs at large scales. In both cases, the first step involves dissolving of the drug in the molten lipid. In hot homogenization, homogenization is applied to the pre-emulsion at a higher temperature than the melting point of lipid. In contrast, in cold homogenization, homogenization of suspension is performed at room temperature.^118,119^

###  Solvent emulsification–diffusion method

 In this method, the polymer or lipid is dissolved in an organic solvent and then emulsified into an aqueous phase containing an emulsifier. Finally, the solvent is evaporated under a vacuum to form polymeric or lipid-based NPs. The advantage of this method over the homogenization method is the lack of high temperature, so it is a suitable method for formulating temperature-sensitive drugs. However, organic solvents may cause toxicological problems.^120,121^

###  Co-precipitation method

 Co-precipitation is the most used method for the preparation of metal oxide and core-shell NPs. It is a cost-effective, fast, straightforward, and easily transposable on a larger scale method for industrial applications. This method gives nanomaterials via high purity and doesn’t require high pressure or temperature and hazardous organic solvents.^122^

###  Phase coacervation

 Coacervation is one of the common methods of microencapsulation and is divided into two categories: simple and complex. In simple coacervation, a colloidal solute such as ethyl cellulose or chitosan is used, while in the case of complex coacervation, a polymer solution is prepared by the interaction between two oppositely charged agents such as gelatin and chitosan. Generally, this method involves the phase-separation of two separate liquid phases to form a polymer-rich phase (coacervate) and a polymer-depleted phase (equilibrium solution).^123,124^

###  Salting out method

 Both the drug and polymer are first dissolved in a solvent in this method. Then, the solubility of the polymer is reduced by adding an electrolyte, and as a result, it precipitates and encapsulates the drug. This technique is primarily used for the preparation of nanospheres.^125,126^

###  Supercritical fluid-based methods

 The supercritical fluid technique with the potential to produce NPs with a narrow size distribution without solvent residues in the final product is considered an essential tool for preparing a wide range of biomedical nanomaterials. Carbon dioxide and water are most commonly used supercritical solvents in this method.^127^ The basis of this method is the dissolution of the drug and carrier materials (e.g., polymer) in the supercritical solvent at critical temperature and pressure and then its expansion by spraying in the expansion chamber at lower pressures, which leads to the deposition of materials and the formation of NPs.^128^

###  Nanoprecipitation technique 

 Nanoprecipitation techniques, also called solvent displacement methods, were developed by Fessi et al.^129^ Usually, in this method, the polymer and drug are dissolved in a water-miscible solvent and then added to a non-solvent. The solubility of the polymer decreases as soon as it enters the nonsolvent and the polymer precipitates encapsulate the drug. The presence of an emulsifier or stabilizer, such as poloxamers is vital to avoid the aggregation of NPs during the nanoprecipitation process.^130^

###  Self-assembly methods

 Self-assembly is the spontaneous arrangement of individual units to create well-defined structures, which is more suitable for preparing two-dimensional nanostructures such as nanosheets. Self-assembly can occur under the influence or in the absence of external intervention, which is called dynamic and static processes, respectively.^131,132^

## Clinical trials and FDA-approved herbal drug delivery nanoformulations

 Cosmetochem Company specialized in the production of a range of botanical extracts in a liposomal powder named Liposome Herbasec^®^. Similarly, a line of Phytosome^®^ technology-based products has been developed and commercialized by the Indena Company. Both liposomal and phytosomal NPs are very efficient penetration enhancers, so they are used as drug carriers for skin with the ability to increase the bioavailability of plant extracts.^15,133^

 In addition, different companies have offered various nanoformulations of anticancer phytomedicines. A summary of anticancer nanophytomedicines, which have entered clinical trials and have also been approved by the FDA, is given in Table 4.

**Table 4:** Clinical trials and FDA-approved anticancer nanophytomedicines

**Phytomedicine**	**Brand name**	**Nanocarrier**	**FDA approved**	**Clinical trials (phase)**	**Govt. clinical trials**
Docetaxel	DoceAqualip	Lipid nanosuspension	Approved in India	I/II/ III	NCT01957995 NCT03671044
	SYP-0709	Polymeric NPs	-	I	NCT02274610 NCT01103791
	LE-DT/ ATI-1123	Liposome	-	I/II	NCT01151384
	CriPec^®^ docetaxel/ CPC634	CriPec NPs	-	I/II	NCT02442531 NCT03742713 NCT03712423
	Docetaxel-PM/ SYP-0704A/ NANOXEL- M	Polymeric micelle	-	II/III	NCT02639858 NCT02982395 NCT03585673
Irinotecan	Onivyde^®^	Liposome	Yes	-	NCT00702182 NCT01494506 ChiCTR-IPR- 15005856
Vincristine	Marqibo^®^	Liposome	Yes	-	-
Vinorelbine tartrate	Navelbine/ NanoVNB^®^	Liposome	Yes	-	NCT03518606 NCT02925000
Curcumin	IMX-110	Curcumin/doxorubicin- encapsulating nanoparticle	Yes	I/II	NCT03382340
	Lipocurc^TM^	Liposome	-	I/II	NCT02138955
Camptothecin	CRLX101/ NLG207	Polymeric nanoparticle	-	I/II	NCT02010567 NCT01380769 NCT01612546
Paclitaxel	NK105	Micellar nanoparticle	-	III	NCT01644890
	Genexol-PM/ IG-001/ Cynviloq	Polymeric micelle	-	I/II/ III/IV	NCT03618758
	Lipusu^®^	Liposome	-	I/II/ III/IV	NCT02142790 NCT02996214
	Abraxane^®^	Albumin-stabilized nanoparticle	Yes	-	NCT02555696 NCT02151149

## Conclusion

 Despite the potential use of plant-derived drugs in the treatment of various diseases, they have considerable limitations due to their high molecular weight, high required dose, poor solubility, and high toxicity. Novel nanotechnology-based drug delivery systems, including polymeric, lipid, and inorganic nanocarriers are beneficial in overcoming these limitations. Nanocarriers containing herbal medicines provide benefits such as increased therapeutic efficacy and bioavailability. Today, many herbal and plant-derived nanoformulations have been approved by the FDA, and many clinical studies are underway in this field.

## Acknowledgments

 The figures were created with Biorender.com.

## Competing Interests

 All authors declare that they have no conflicts of interest.

## Ethical Approval

 Not applicable.
